# Supplementary material for: Combination Therapy of Chloroquine and C2-Ceramide Enhances Cytotoxicity in Lung Cancer H460 and H1299 Cells
Source: Cancers (Basel). 2019 Mar 15;11(3):370. doi: 10.3390/cancers11030370 (PMC6468447; doi:10.3390/cancers11030370)
Supplement: Supplementary file 1 [file cancers-11-00370-s001.pdf]

Supplementary material: Combination Therapy of Chloroquine and C_2-Ceramide Enhances Cytotoxicity in Lung Cancer H460 and H1299 Cells

Han-Lin Chou, Yi-Hsiung Lin, Wangta Liu, Chang-Yi Wu, Ruei-Nian Li, Hurng-Wern Huang, Chi-Hsien Chou, Shean-Jaw Chiou and Chien-Chih Chiu

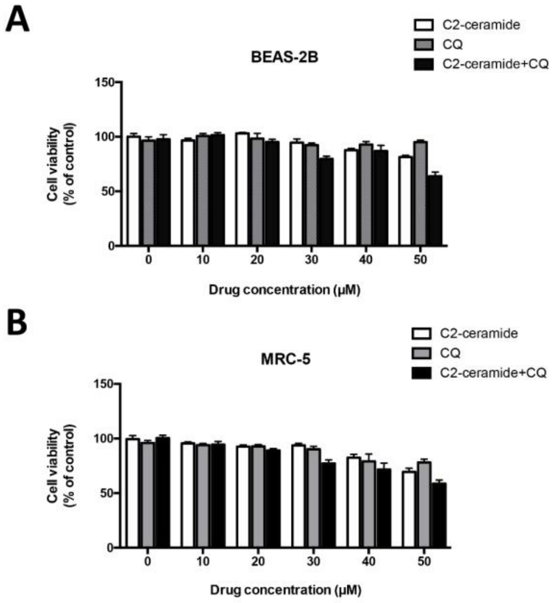

Figure S1. The cell viability determination of normal lung cells with C_2-ceramide and CQ cotreatment. The cytotoxicity of C_2-ceramide and CQ combined treatment was investigated in normal lung cells including (**A**) BEAS-2B and (**B**) MRC-5. The cells were incubated with the indicated concentrations of drugs for 24 hr. The equal concentration of two drugs was used in the group of combined treatment.

© 2019 by the authors. Licensee MDPI, Basel, Switzerland. This article is an open access article distributed under the terms and conditions of the Creative Commons Attribution (CC BY) license (http://creativecommons.org/licenses/by/4.0/).
